# Cross-matrix multi-omics profiling identifies host–microbe interactions and diagnostic signatures in bovine subclinical mastitis

**DOI:** 10.3389/fmicb.2025.1613949

**Published:** 2025-08-05

**Authors:** Yuqiong Li, Xiulan Xie, Youli Yu, Song Hua, ZhuMing Zhang, Zhengwei Zhao, Haihui Gao, Chenglian Zhang, Meizhou Huang

**Affiliations:** ^1^Institute of Animal Science, Ningxia Academy of Agricultural and Forestry Sciences, Yinchuan, China; ^2^College of Veterinary Medicine, Northwest A&F University, Xianyang, China; ^3^College of Animal Science and Technology, Ningxia University, Yinchuan, China; ^4^Ningxia Hui Autonomous Region Animal Disease Prevention and Control Center, Yinchuan, China; ^5^Metabolic Hepatobiliary and Pancreatic Diseases Key Laboratory of Luzhou City, Academician (Expert) Workstation of Sichuan Province, Department of General Surgery (Hepatopancreatobiliary Surgery), The Affiliated Hospital of Southwest Medical University, Luzhou, China

**Keywords:** subclinical mastitis, multi-omics approach, biomarkers, metabolomics, microbiome

## Abstract

Subclinical mastitis (SCM) is a widespread but frequently undetected condition in dairy cows, leading to reduced milk quality and compromised animal health. This study utilizes an integrated multi-omics strategy encompassing metabolomics and microbiome analyses to investigate the systemic effects of SCM across four biological matrices: blood, milk, feces, and rumen fluid. Our findings reveal significant alterations in hematological and biochemical parameters, with key biomarkers such as digalacturonic acid and N-ε-methyl-L-lysine indicating systemic metabolic and immune dysregulation. Metabolomic profiling uncovered distinct disease-related metabolic patterns, while 16S rRNA sequencing revealed substantial microbial shifts, particularly involving *Succinivibrio* and *Methanobrevibacter*, which are implicated in carbohydrate fermentation and methanogenesis. Noteworthy correlations between specific metabolites (e.g., ropinirole, arachidonic acid) and microbial genera (e.g., *Succinivibrionaceae UCG-001*, *Alistipes*) highlight the complex host-microbiome-metabolite interplay associated with SCM. These findings provide new insights into the pathophysiology of SCM and identify candidate biomarkers for early detection. The integrative multi-omics approach adopted in this study offers a valuable framework for developing innovative diagnostic and therapeutic strategies to enhance dairy cow health and productivity.

## 1 Introduction

Bovine mastitis, particularly in its subclinical form (SCM), remains a persistent challenge for the global dairy industry due to its high prevalence, asymptomatic nature, and detrimental impact on milk quality, animal welfare, and farm profitability (Demil et al., 2022; Abed et al., 2021; Kumari et al., 2018; Argaw, 2016; Wang et al., 2024). Unlike clinical mastitis, SCM lacks overt clinical signs such as udder swelling or abnormal milk, making early detection difficult (Tommasoni et al., 2023; Ruegg, 2012). Globally, SCM affects approximately 42% of dairy cows (Abed et al., 2021; Tassew et al., 2016; El-Tawab et al., 2016), whereas clinical mastitis occurs in only about 15%, highlighting the need for enhanced surveillance and intervention strategies (Khasapane et al., 2024).

The economic implications of SCM are substantial, as it leads to elevated somatic cell counts (SCC), reduced milk yield, and compromised product safety (Kumari et al., 2018; Wang et al., 2024; Qolbaini et al., 2021). These challenges are further compounded by increasing antimicrobial resistance in common pathogens such as *Staphylococcus aureus* and *Streptococcus agalactiae* (Farabi et al., 2024; Michira et al., 2023; Tassew, 2017; Eleodoro et al., 2022), making SCM increasingly difficult to treat effectively. Addressing SCM is therefore essential not only for improving herd productivity and animal health but also for safeguarding food quality and public health.

Advances in molecular biology and systems-level research have significantly deepened our understanding of host-pathogen interactions in mastitis (Eleodoro et al., 2022; Mudaliar et al., 2017; Zhu et al., 2024). The application of systems biology has uncovered various virulence factors, host genetic susceptibilities, immune system impairments, and molecular mechanisms that contribute to disease progression (Couvillion et al., 2023; Tong et al., 2019). Notably, predictive metabolomic profiling has identified serum biomarkers such as lysine, leucine, isoleucine, and kynurenine that can distinguish SCM cows from healthy animals weeks before parturition, providing opportunities for early intervention (Zandkarimi et al., 2018; Haxhiaj et al., 2022; Wang et al., 2021b).

Multi-omics approaches, integrating metabolomics, transcriptomics, proteomics, and microbiome analyses, provide a holistic perspective on complex biological processes and disease mechanisms (Alessandri et al., 2023a; Xi et al., 2017; Thomas et al., 2016; Wang et al., 2022). The use of diverse biological samples including cells, tissues, and bodily fluids enhances the detection of disease-specific biomarkers and provides insights into systemic changes associated with pathogenesis. Lipidomics analyses have identified over 600 altered lipid species in SCM milk, particularly in infections caused by non-*aureus* Staphylococci, which may serve as diagnostic indicators (Wang et al., 2020; Zhu et al., 2023). Additionally, subclinical inflammation is associated with elevated levels of serum NEFA, LDH, and globulins, indicating both metabolic stress and inflammatory responses (Ceciliani et al., 2020; Tang et al., 2024).

SCM is a multifactorial disease influenced by environmental conditions, pathogen load, immune competence, and the status of the gut and rumen microbiota (Wang et al., 2021a; Saleh et al., 2022; Alessandri et al., 2023b). While microbial invasion initiates mammary gland inflammation, the broader systemic progression of SCM is modulated by stress, nutrition, hygiene, and immune function. Studies have also shown that poor milking hygiene, inadequate farm cleanliness, and substandard housing conditions significantly increase SCM prevalence (Wang et al., 2021c; Wang et al., 2022). To address this complexity, we employed a comprehensive multi-omics framework that integrates analyses of from blood, milk, feces, and rumen fluid. This innovative design enables a multi-dimensional exploration of the metabolic, microbial, and immunological factors underpinning SCM, paving the way for more effective detection and prevention strategies in dairy herd health management.

## 2 Materials and methods

### 2.1 Chemicals and materials

All solvents used for liquid chromatography-mass spectrometry (LC-MS), including methanol, acetonitrile (ACN), and isopropanol, were of LC-MS grade and obtained from Fisher Scientific (Loughborough, United Kingdom). LC-MS additives such as formic acid and ammonium acetate were purchased from Sigma-Aldrich (Madrid, Spain). The ACQUITY UPLC BEH Amide column was supplied by Waters (Milford, MA, United States). Sterile vacuum blood collection tubes were provided by BD Medical Devices Shanghai Co., Ltd. (Shanghai, China).

### 2.2 Animal selection and sample collection

This prospective, single-location study was conducted at a commercial dairy farm in western Yinchuan with approximately 4,000 Holstein cows. All animals were managed under consistent conditions, including a standardized total mixed ration (TMR) formulated according to NRC guidelines, uniform free-stall housing, and regular health monitoring. Milking procedures were fully automated and followed routine pre- and post-milking sanitation protocols. The farm also implemented a centralized Dairy Herd Improvement (DHI) system for monthly monitoring of milk production and health parameters, including somatic cell counts (SCC).

The average annual incidence of subclinical mastitis (SCM) on this farm was 30.4%, based on DHI records over the past 3 years. This rate is within the expected range for large-scale dairy operations in the region. SCM diagnosis was determined by SCC values greater than 200,000 cells/mL, in line with international thresholds. Measurements were collected monthly through the DHI system.

Ethical approval for animal handling and sampling was obtained from the Animal Care and Use Committee of the Ningxia Academy of Agricultural and Forestry Sciences (Approval No. 2022-06). Cows diagnosed with SCM met the following criteria: (1) no visible clinical signs, (2) SCC >200,000 cells/mL in the most recent DHI report, and (3) no recent use of antibiotics or presence of systemic illness. Healthy controls had SCC <200,000 cells/mL and showed no signs of mastitis or other disease.

A total of 20 cows were selected—10 with SCM and 10 healthy controls—matched for age and parity. Each cow provided one sample from blood, milk, feces, and rumen fluid, resulting in 80 biological samples. The sample size was informed by previous multi-omics studies in dairy science and was considered sufficient to detect meaningful differences in both metabolomic and microbiome profiles (Wang et al., 2021a). Samples collected included blood, milk, feces, and rumen fluid. Blood was collected aseptically from the jugular vein and processed for serum separation. Milk samples were obtained during mid-milking using sterile containers after teat disinfection. Fecal samples were collected immediately after defecation using sterile tools. Rumen fluid was aspirated under veterinary supervision using a rumen cannula. All samples were stored at 4°C during transport and processed within 4 h.

### 2.3 Hematological and biochemical analysis

Blood samples were divided into two aliquots: one for complete blood count (CBC) using an automated veterinary hematology analyzer (Mindray BC-5000 Vet, China) and another for serum biochemical analysis. CBC parameters included white blood cell (WBC) count, red blood cell (RBC) count, hemoglobin (Hb), hematocrit (HCT), and platelet count (PLT). Serum biochemical analyses were performed using standardized clinical chemistry analyzers.

Milk quality parameters including fat, protein, lactose, and milk urea nitrogen were quantified using Fourier-transform infrared (FTIR) spectroscopy (MilkoScan FT120, Denmark). Somatic cell counts were measured via flow cytometry using a Fossomatic FC analyzer (Denmark). All instruments were routinely calibrated with certified reference materials.

### 2.4 Metabolomics profiling via LC-MS

Sample preparation protocols were optimized for each matrix. Milk was defatted by cold centrifugation, while feces and rumen fluid were homogenized and filtered. Proteins were precipitated using a methanol: acetonitrile mixture. Extracted metabolites were analyzed using ultra-high-performance liquid chromatography (UHPLC) coupled with time-of-flight mass spectrometry (TOF-MS). Data were converted to mzXML format using ProteoWizard and processed using XCMS and CAMERA for peak detection, alignment, and annotation. Normalization was performed prior to statistical analysis. Multivariate analyses including principal component analysis (PCA) and partial least squares-discriminant analysis (PLS-DA) were conducted using R software. Metabolic pathway enrichment was performed using KEGG database annotations. Detailed methodology is provided in the Supplementary material.

### 2.5 16S rRNA gene sequencing for bovine fecal and rumen microbiome analysis

Microbial DNA was extracted from fecal and rumen samples using mechanical and chemical lysis protocols. DNA quality and quantity were assessed with spectrophotometry and fluorometry. The V3–V4 region of the 16S rRNA gene was amplified, purified, and sequenced on an Illumina platform. Bioinformatic analysis included quality filtering, operational taxonomic unit (OTU) clustering, taxonomic classification, and alpha/beta diversity evaluation. Rarefaction curves were generated to ensure consistent sequencing depth and sample coverage across groups. Detailed methodology is provided in the Supplementary material.

### 2.6 Integrated analysis of metabolomic and microbiome interactions

Integrated analyses were performed to correlate metabolomic and microbiome data. Normalized datasets were subjected to correlation analysis (Pearson or Spearman), network construction, and pathway enrichment using KEGG and Reactome databases. Feature selection and statistical validation were conducted using permutation testing and false discovery rate (FDR) correction to ensure robustness.

### 2.7 Statistical analyses

All statistical analyses were performed using SPSS version 22.0 (IBM Corp., Armonk, NY, United States). Normality was assessed using the Shapiro–Wilk test. Parametric data were expressed as mean ± SEM and analyzed using one-way ANOVA, followed by Tukey’s HSD test for multiple comparisons. Statistical significance was defined as *p* < 0.05. For microbiome data, OTUs were clustered at 97% sequence similarity using the UPARSE algorithm and annotated using the SILVA reference database (v138). Taxonomic profiles were summarized at the genus level for downstream analyses, including diversity metrics and metabolite correlation. Prior to statistical testing, low-abundance features were filtered to improve analytical robustness. Only OTUs with a relative abundance of ≥0.1% present in at least 30% of samples were retained. This filtering strategy was applied uniformly to reduce statistical noise and improve the biological interpretability of microbial patterns associated with subclinical mastitis.

## 3 Results

### 3.1 Hematological and biochemical alterations in subclinical mastitis

Cows with SCM exhibited a significant elevation in white blood cell (WBC) counts, particularly in basophils (Table 1), indicating an activated immune response. These findings align with the subclinical inflammatory nature of SCM, differentiating it from clinical mastitis, which is typically characterized by more pronounced neutrophil dominance. Other immune cell subsets, including neutrophils, lymphocytes, monocytes, and eosinophils, did not display statistically significant differences (Table 1), supporting the notion of milder systemic immune activation in SCM.

**Table 1:** Comparison of hematological parameters between healthy and SCM dairy cows.

Parameter	Health group	SCM group	*p*-value
WBC (10^9^/L)	6.97 ± 1.05	10.03 ± 2.14	0.008
Neu # (10^9^/L)	2.79 ± 0.53	3.20 ± 1.46	0.515
Lym # (10^9^/L)	3.70 ± 0.65	3.17 ± 0.82	0.211
Mon # (10^9^/L)	0.36 ± 0.07	0.48 ± 0.16	0.115
Eos # (10^9^/L)	0.08 ± 0.04	0.14 ± 0.25	0.560
Bas # (10^9^/L)	0.03 ± 0.01	0.06 ± 0.01	<0.001
Neu % (%)	40.03 ± 4.64	43.71 ± 14.09	0.531
Lym % (%)	52.99 ± 3.87	46.13 ± 10.98	0.160
Mon % (%)	5.26 ± 1.41	7.26 ± 3.40	0.189
Eos % (%)	1.14 ± 0.59	1.84 ± 2.90	0.553
Bas % (%)	0.51 ± 0.11	0.70 ± 0.08	0.004
RBC (10^12^/L)	7.63 ± 1.11	7.03 ± 1.02	0.316
HGB (g/L)	101 ± 9.73	96.71 ± 10.97	0.454
HCT (%)	40.96 ± 3.57	39.41 ± 4.63	0.499
MCV (fL)	54.20 ± 5.24	56.39 ± 4.76	0.430
MCH (pg)	13.36 ± 1.08	13.80 ± 1.00	0.442
MCHC (g/L)	246.57 ± 4.89	245.14 ± 3.85	0.556
RDW-CV (%)	20.30 ± 0.58	20.37 ± 0.85	0.858
RDW-SD (fL)	31.67 ± 3.25	33.07 ± 3.50	0.454
PLT (10^9^/L)	327.29 ± 116.00	458.57 ± 351.78	0.378
MPV (fL)	6.76 ± 0.51	6.64 ± 0.68	0.729
PDW (fL)	15.16 ± 0.30	15.27 ± 0.24	0.449
PCT (%)	0.219 ± 0.07	0.312 ± 0.26	0.386

In addition, notable metabolic changes were observed. Milk from SCM-affected cows exhibited significantly reduced fat and lactose content, along with increased protein levels (Table 2), suggesting metabolic reprogramming associated with the inflammatory process. While changes in urea, total solids, red blood cell indices, and platelet parameters were noted, they did not reach statistical significance (Table 2).

**Table 2:** Comparison of major milk constituents between healthy and SCM dairy cows.

Parameter	Control (mean ± SD)	Model (mean ± SD)	*p*-value
FatB(T)	2.16 ± 0.21	1.42 ± 0.24	0.00005
Prot(T)	3.18 ± 0.12	3.57 ± 0.31	0.01394
Lact(T)	4.41 ± 0.35	3.74 ± 0.15	0.00160
TS(T)	10.84 ± 0.73	10.15 ± 0.66	0.08643
Cells	17.71 ± 16.40	1983.29 ± 382.47	0.00001
Urea	7.24 ± 0.62	6.03 ± 1.64	0.10505

### 3.2 Metabolomic profiling reveals key biomarkers and systemic disturbances

**Figure 1:**
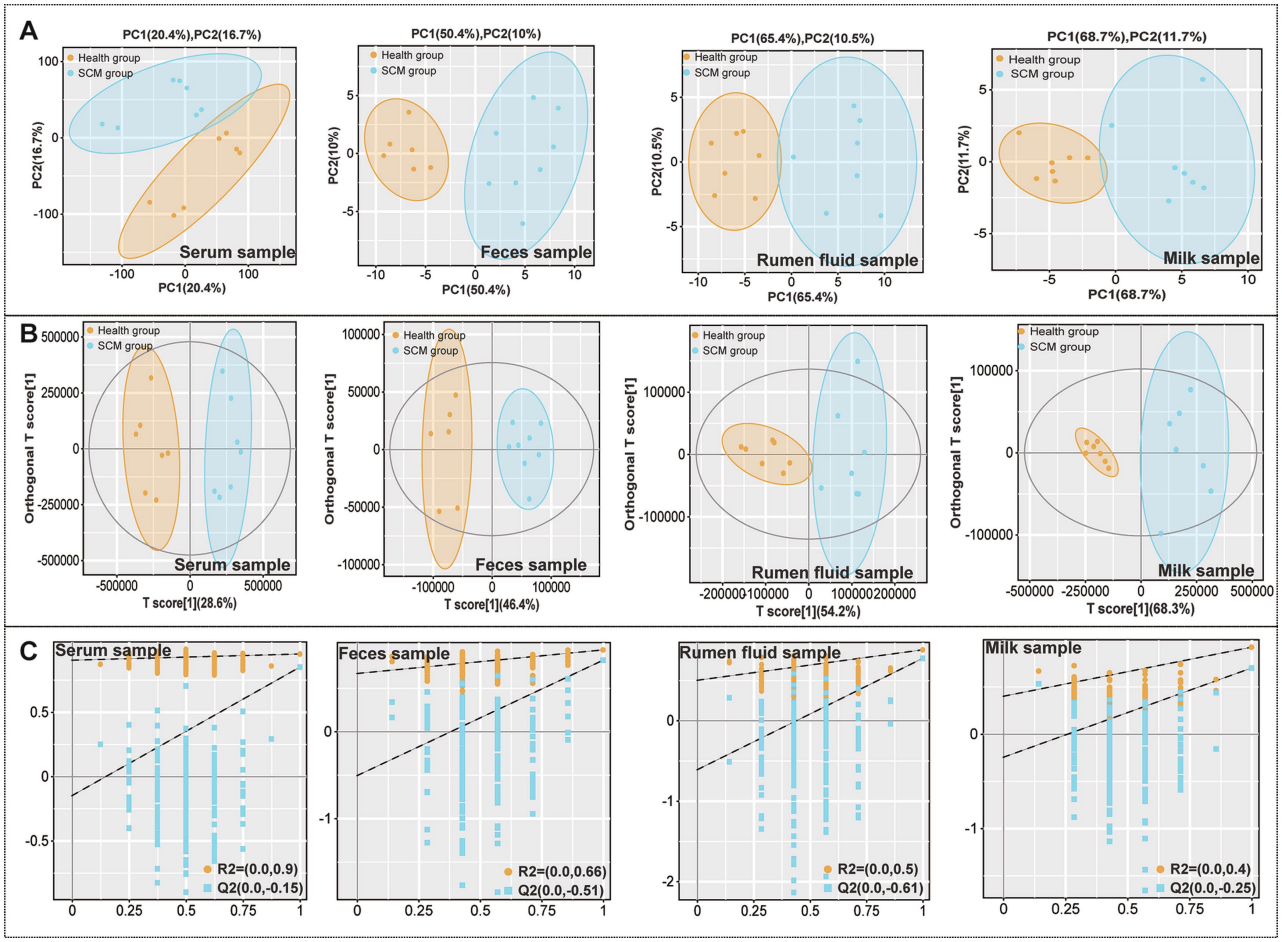
Metabolic alterations across sample types in subclinical mastitis (SCM) and healthy dairy cows. **(A)** Principal component analysis (PCA) score plots visualizing the separation of metabolic profiles in serum, feces, rumen fluid, and milk between healthy and SCM cows. **(B)** Orthogonal partial least squares discriminant analysis (OPLS-DA) score plots showing discriminatory metabolic signatures across the same sample types. **(C)** Permutation test results confirming the statistical validity and robustness of the OPLS-DA models for each matrix.

**Figure 2:**
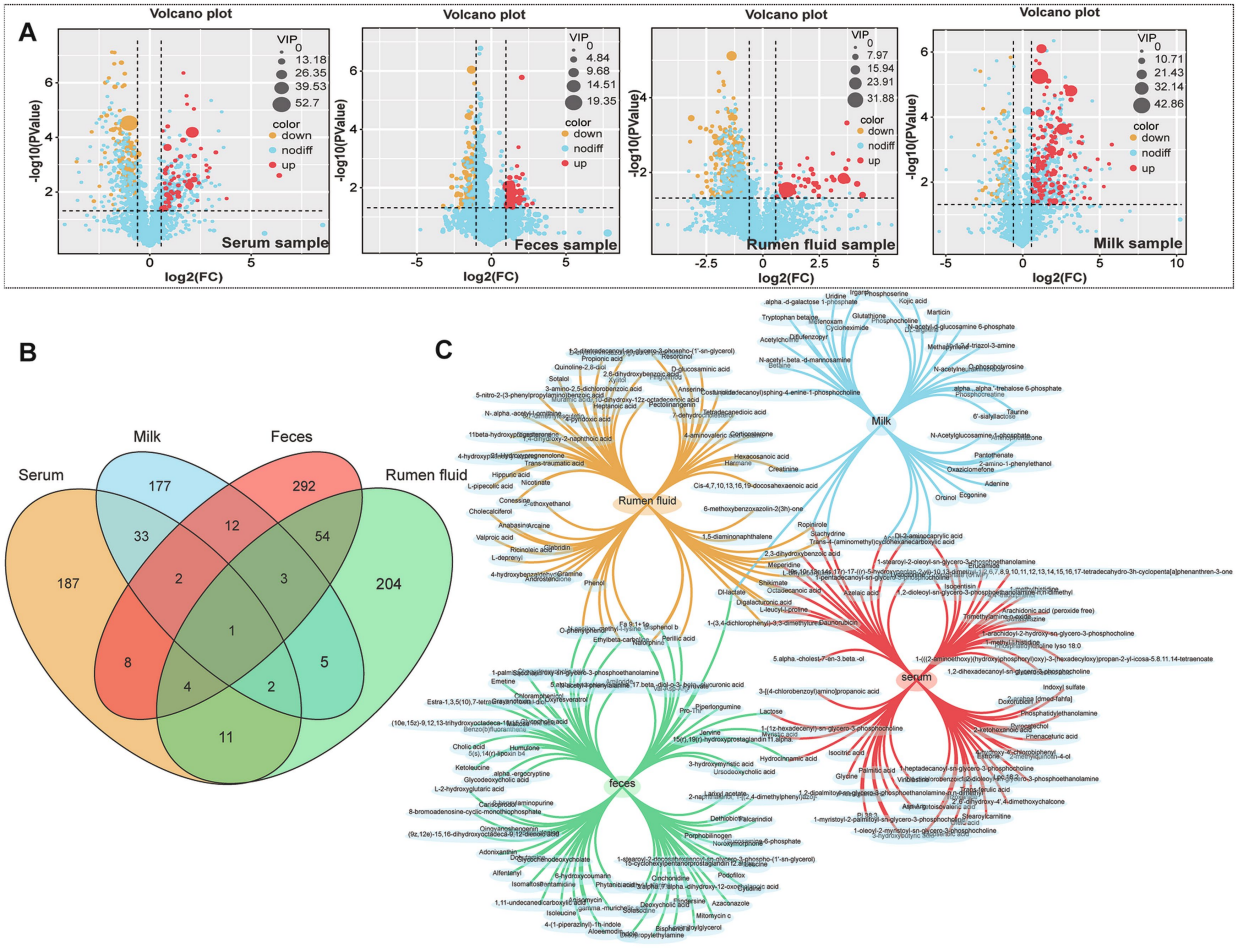
Identification and distribution of differential metabolites between healthy and SCM cows. **(A)** Volcano plots presenting significant differential metabolites (cutoff: |log2FC| >1, *p* < 0.05) in serum, feces, rumen fluid, and milk. **(B,C)** Venn diagrams illustrating the overlap and uniqueness of differential metabolites across the four biological matrices.

Comprehensive metabolomic profiling via LC-MS identified substantial alterations in metabolite composition across serum, milk, rumen fluid, and fecal samples. Orthogonal projections to latent structures discriminant analysis (OPLS-DA) confirmed clear group separation and robust differential metabolite identification (Figure 1). A total of 248 differential metabolites were identified in serum, 235 in milk, 284 in rumen fluid, and 376 in feces (Figures 2A–C). To validate the observed group separation and further strengthen the statistical interpretation, we performed PERMANOVA (Permutational Multivariate Analysis of Variance) using Bray–Curtis dissimilarity and 999 permutations. Significant differences between subclinical mastitis (SCM) and healthy cows were observed in multiple sample types, including serum (*p* = 0.019), milk (*p* = 0.007), and feces (*p* = 0.022). These results provide statistical support for the metabolomic group differences and are presented in Supplementary Table S1.

Cross-sample comparisons revealed shared metabolites across matrices, including digalacturonic acid, lactose, and N-ε-methyl-L-lysine, which were consistently altered in SCM-affected cows (Table 3). Elevated levels of lactose and N-ε-methyl-L-lysine were found in both serum and milk (VIP >1.0; *p* < 0.0001), while digalacturonic acid was notably increased in fecal samples (Table 3), corroborating previous reports of its association with gut inflammation.

**Table 3:** The potentially differential metabolites in SCM dairy cows.

Sample type	Differential metabolites	VIP	log2FC (SCM/health)	*p*-value
Serum	Lactose	11.03	2.3	0.006
Serum	N-epsilon-methyl-L-lysine	1.01	0.92	0.002
Serum	Acetylcarnitine	16.68	2.01	0.016
Serum	Ropinirole	1.41	2.62	0.0007
Milk	Digalacturonic acid	1.10	2.31	0.0006
Milk	Lactose	16.58	0.98	0.00004
Milk	N-epsilon-methyl-L-lysine	2.17	1.10	0.00001
Milk	Ropinirole	2.06	5.02	0.0015
Feces	Digalacturonic acid	1.86	−1.00	0.005
Feces	Lactose	9.02	1.75	0.011
Feces	N-epsilon-methyl-L-lysine	6.53	0.86	0.029
Rumen fluid	N-epsilon-methyl-L-lysine	3.65	−1.26	0.0004
Rumen fluid	Ropinirole	2.16	−0.74	0.04907

Other metabolites of interest included acetylcarnitine and ropinirole, detected across multiple sample types, indicating their potential role in energy metabolism and neuromodulation in the context of SCM.

### 3.3 Metabolic pathway disruptions associated with SCM

**Figure 3:**
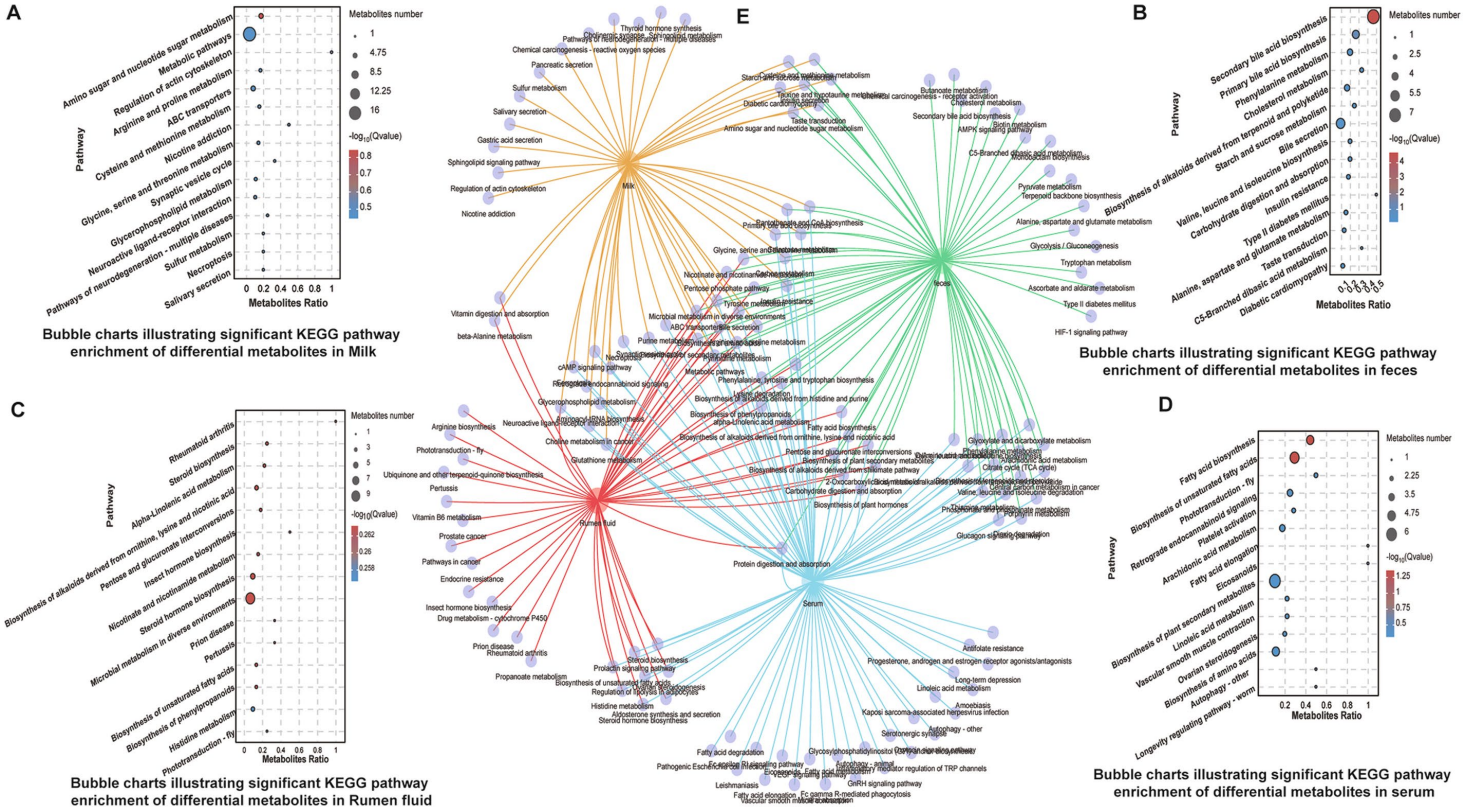
KEGG pathway enrichment analysis of identified differential metabolites. **(A–D)** Bubble charts indicating significantly enriched KEGG pathways in serum, feces, rumen fluid, and milk, respectively. **(E)** Composite Venn diagram comparing enriched pathways across the four sample types, highlighting shared and unique metabolic routes.

Pathway enrichment analysis revealed that differentially expressed metabolites were significantly enriched in amino acid biosynthesis, pyrimidine metabolism, microbial metabolism, and ABC transporter pathways across all matrices (Figures 3A–D). Unique enrichment was observed in specific compartments: unsaturated fatty acid biosynthesis in serum, galactose metabolism in milk and serum, steroid biosynthesis in rumen fluid, and bile acid biosynthesis in feces (Figure 3E). These findings reflect compartment-specific metabolic responses to subclinical inflammation and microbial perturbation.

### 3.4 Microbiota alterations in rumen and feces

**Figure 4:**
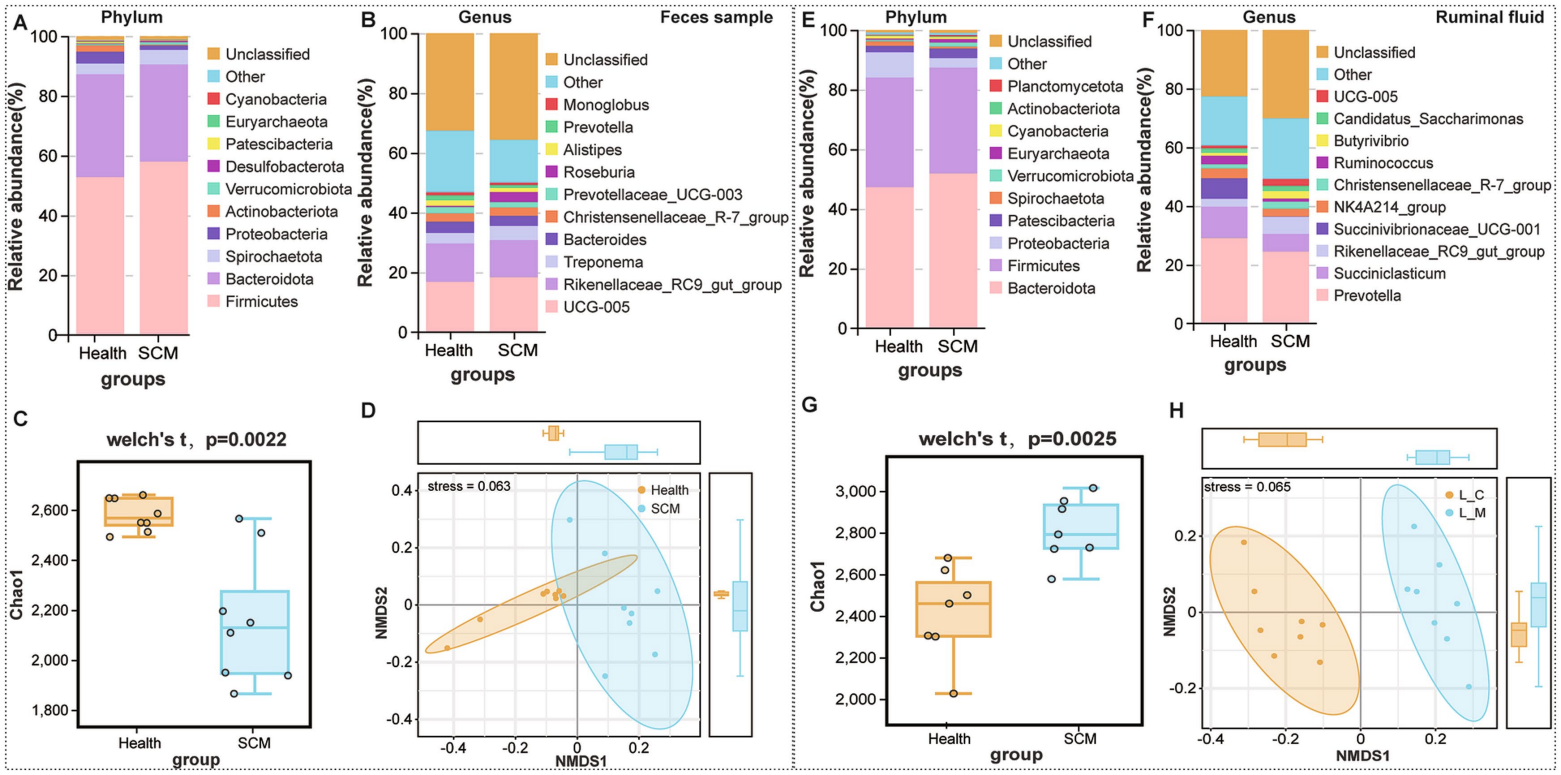
Microbiota composition and diversity shifts in feces and rumen fluid between healthy and SCM cows. **(A,B)** Stacked bar plots depicting microbial composition at the phylum and genus levels in fecal samples. **(C)** Alpha diversity analysis of fecal microbiota using the Chao1 index. **(D)** Non-metric multidimensional scaling (NMDS) analysis reflecting beta diversity in fecal microbiota. **(E,F)** Microbial composition in rumen fluid samples at phylum and genus levels. **(G)** Alpha diversity analysis in rumen fluid using Chao1 index. **(H)** NMDS plots displaying beta diversity in rumen microbiota.

16S rRNA sequencing revealed notable microbial community shifts in SCM cows. In rumen fluid, relative abundances of *Spirochaetota* increased, while *Euryarchaeota* and *Planctomycetota* decreased. In feces, *Actinobacteriota*, *Patescibacteria*, and *Cyanobacteria* were significantly reduced (Figures 4A,B). Alpha diversity analysis showed a significant decrease in rumen microbial richness in SCM cows (*p* < 0.05), as reflected by Chao1 (Figure 4G). Fecal microbial diversity showed a similar downward trend (Figure 4C). Beta diversity analyses further underscored the distinct clustering of microbial communities in SCM cows (Figures 4D,H).

At the genus level, 21 genera were differentially abundant in rumen fluid (Figures 4E,F), including *Succiniclasticum* and *Succinivibrio*, while 13 genera, such as *Alistipes* and *Methanobrevibacter*, were altered in feces. Several genera including *Succinivibrionaceae UCG-001*, *Alistipes*, and *Ruminococcus torques* group were identified as core taxa altered in both rumen and feces, though with opposing trends in abundance.

**Figure 5:**
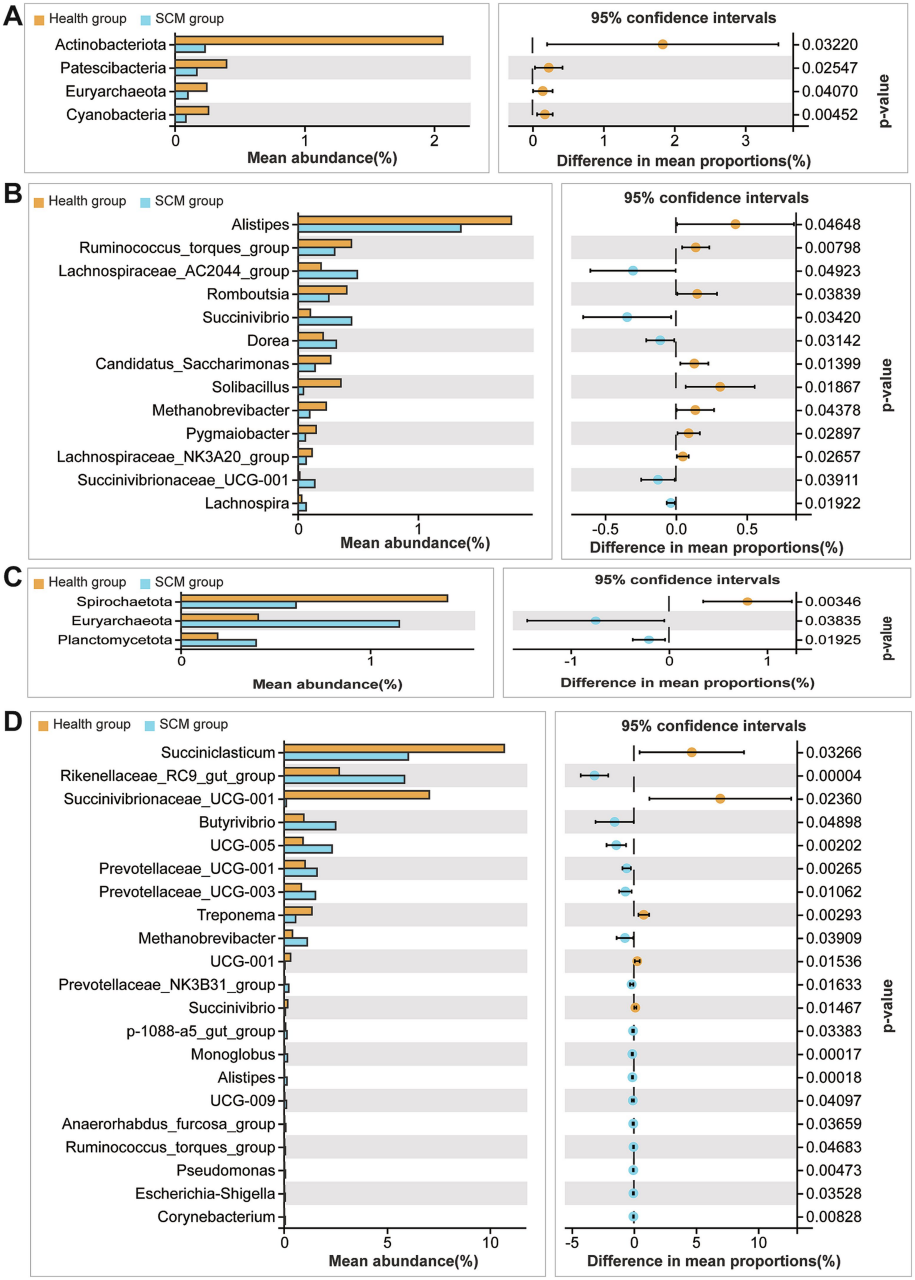
Taxonomic comparison of microbial species between healthy and SCM cows. **(A,B)** Differential abundance of microbial taxa in fecal samples at phylum and genus levels. **(C,D)** Differential microbial species identified in rumen fluid at phylum and genus levels.

**Figure 6:**
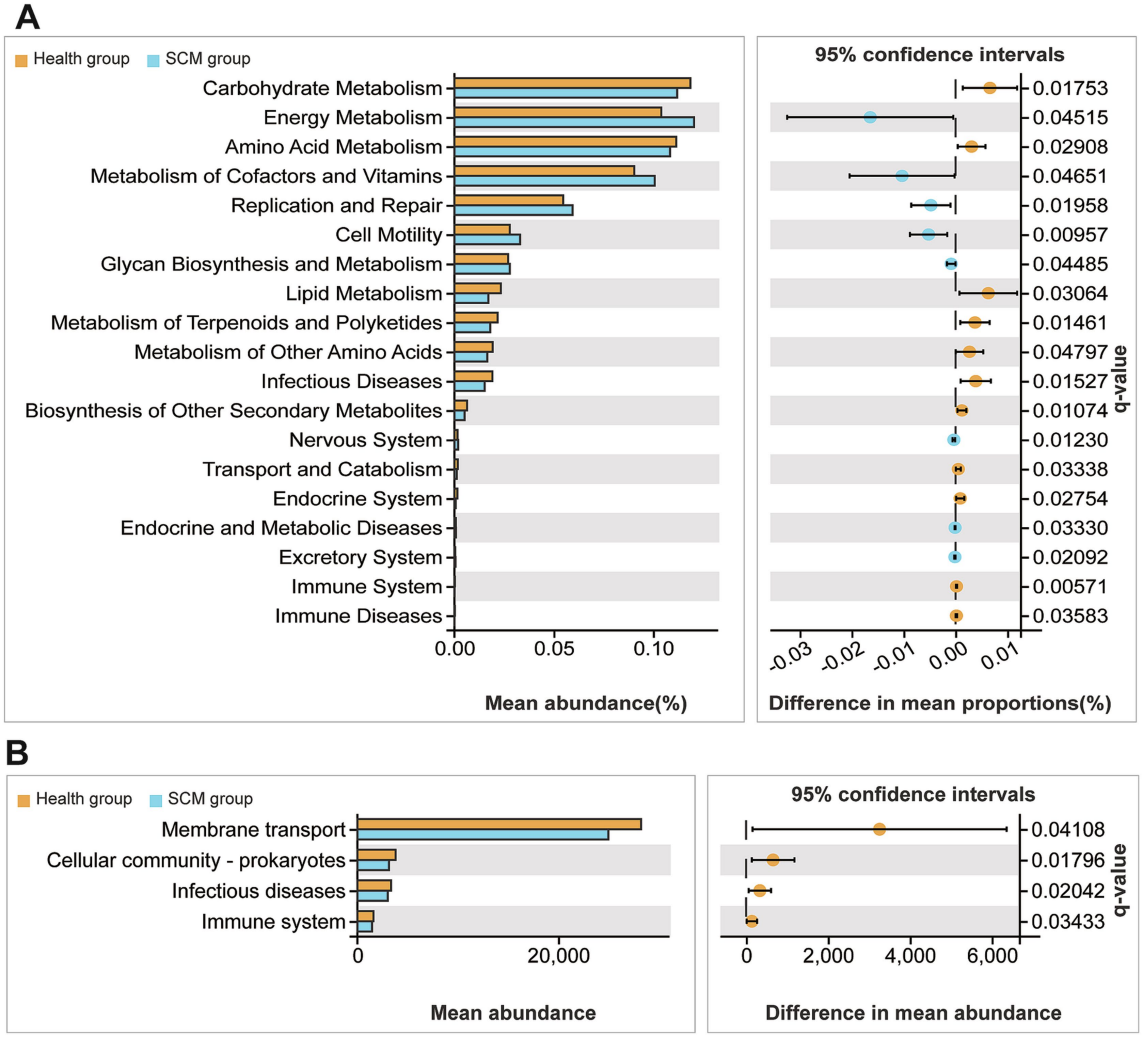
Functional profiling of differential microbial taxa in feces and rumen fluid. **(A,B)** KEGG-based functional prediction analyses revealing enriched metabolic and signaling pathways among the differential microbiota from SCM vs. healthy cows.

Predicted functional profiling using PICRUSt2 suggested that microbial shifts in SCM were associated with immune-related pathways, including cytokine signaling and antigen processing. These predictions are inferential and based on 16S taxonomic data, not direct functional measurements (Figures 5, 6).

### 3.5 Metabolite-microbiome correlations

**Figure 7:**
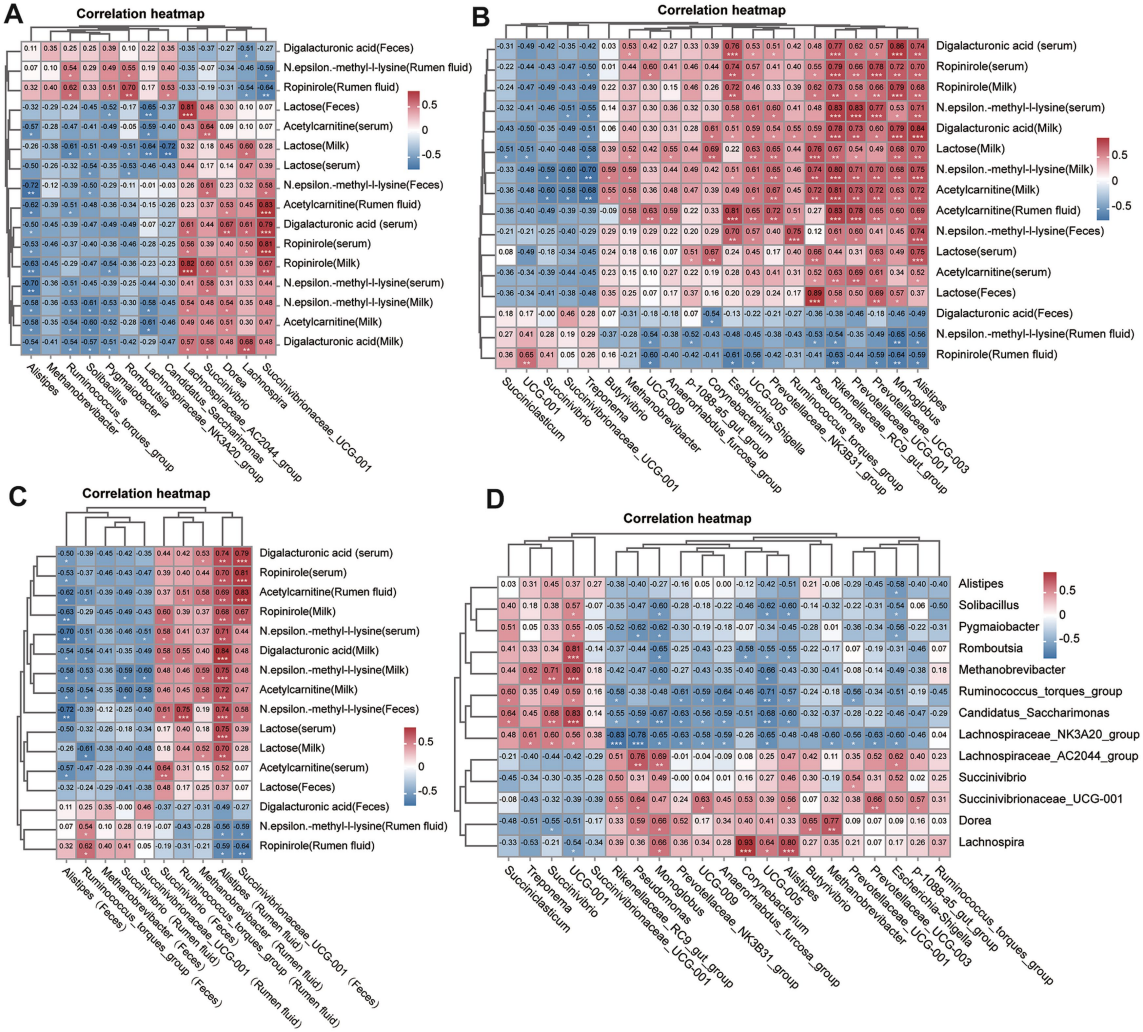
Correlation analyses between key metabolites and microbial taxa. **(A)** Heatmap showing Spearman correlations between significantly altered fecal microbiota and differential metabolites. **(B)** Correlation heatmap between rumen microbiota and corresponding metabolites. **(C)** Cross-matrix correlations between fecal and rumen microbial taxa. **(D)** Global correlation overview summarizing associations between all significant microbiota and metabolites across sample types. *p*-values were adjusted using the Benjamini–Hochberg method, and the significance threshold was adjusted *p* < 0.05.

Correlation analysis between differential metabolites and microbial genera revealed several significant associations. Correlation analysis between differential metabolites, alpha diversity indices and microbial genera was conducted using Spearman correlation with FDR-adjusted *p*-values. Associations were visualized in heatmaps (Figure 7), and significance was defined as adjusted *p* < 0.05. A negative correlation was observed between alpha diversity and inflammatory metabolites such as digalacturonic acid. Ropinirole showed positive correlation with Succinivibrionaceae UCG-001; Alpha diversity indices, particularly Chao1, were negatively correlated with inflammatory metabolites such as N-ε-methyl-L-lysine and digalacturonic acid, suggesting that reduced microbial richness may drive metabolic dysregulation in SCM. Ropinirole and arachidonic acid positively correlated with *Succinivibrionaceae UCG-001*, while *acetylcarnitine* showed similar correlations in rumen fluid (Figure 7). Conversely, N-ε-methyl-L-lysine exhibited a negative association with *Alistipes* in multiple sample types. These correlations point to potential metabolite-microbiota interaction networks that may underlie SCM pathology and serve as targets for biomarker discovery.

## 4 Discussion

Subclinical mastitis (SCM) imposes substantial economic and welfare burdens on the dairy industry due to its asymptomatic nature, delayed detection, and persistent impact on milk yield and quality (Demil et al., 2022; Abed et al., 2021; Kumari et al., 2018). Our study provides an in-depth exploration of the systemic changes associated with SCM using a multi-omics framework that integrates metabolic and microbiome data from multiple biological matrices. This comprehensive approach enhances current understanding of the disease’s pathophysiology and offers valuable targets for early diagnosis and intervention (Tong et al., 2019; Zandkarimi et al., 2018; Haxhiaj et al., 2022; Wang et al., 2021b; Alessandri et al., 2023a).

The observed hematological shifts, including elevated WBC and basophil counts, suggest an ongoing low-grade inflammatory state in SCM-affected cows. Unlike clinical mastitis, where acute inflammation is evident, SCM is marked by subtler yet persistent immune activation. This chronic immunological imbalance likely contributes to metabolic disturbances and altered nutrient partitioning, as evidenced by decreased milk fat and lactose levels alongside increased protein content (Xi et al., 2017; Thomas et al., 2016).

In addition to immune-related changes, nutritional factors have emerged as crucial regulators of the gut and rumen microbiome in ruminants. Variations in dietary fiber type, fermentable carbohydrate content, and inclusion of prebiotics are known to influence microbial community composition and metabolic activity, with implications for host immunity and mammary gland health. For instance, substantial shifts in both intestinal and milk microbial populations have been linked to dietary modifications, underscoring the direct impact of nutrition on microbial dynamics (Alvanou et al., 2024). Evidence also suggests that functional feed additives can positively modulate microbiota-related inflammation. In previous study, dietary inulin was shown to promote the abundance of beneficial microbes and enhance anti-inflammatory metabolic pathways, ultimately leading to a measurable reduction in SCM-associated biomarkers in both feces and milk (Wang et al., 2021a). Furthermore, multi-omics research has indicated that tailored nutritional interventions can alter the milk microbiome structure in ways that may support epithelial integrity and improve resistance to infection (Couvillion et al., 2023).

Although a uniform total mixed ration (TMR) was applied across all animals in this study to minimize dietary variability, differences in individual feed intake behavior, digestive efficiency, or microbiota-host interactions could still account for the microbial and metabolic heterogeneity observed between groups. Particularly noteworthy are the taxa *Succinivibrionaceae UCG-001* and *Alistipes*, both of which are sensitive to substrate availability and dietary shifts. Their differential abundance in SCM cows may therefore reflect an indirect influence of nutrition, even under standardized feeding. Future investigations incorporating dietary manipulation alongside longitudinal microbiome and metabolome profiling will be essential to uncover the causal links between nutrition, microbial balance, and SCM. Through untargeted metabolomic profiling, we identified consistent alterations in specific metabolites such as digalacturonic acid, N-ε-methyl-L-lysine, ropinirole, and acetylcarnitine across serum, milk, feces, and rumen fluid. The reproducibility of these findings across matrices underscores their potential as reliable biomarkers. Additionally, although ropinirole was detected across multiple matrices and confirmed in serum via a targeted LC-MS/MS method, its synthetic nature necessitates caution in interpretation. The presence of ropinirole in 16 out of 20 cows suggests consistent exposure, yet its source remains unclear. Environmental contamination, feed additives, or veterinary drugs may be potential contributors. Until these factors are verified, ropinirole should not be considered a reliable endogenous biomarker for SCM. N-ε-methyl-L-lysine, in particular, emerged as a robust candidate due to its consistent dysregulation, aligning with its known role in immune modulation and inflammation. Similarly, elevated arachidonic acid, a precursor of pro-inflammatory mediators, supports the inflammatory underpinnings of SCM (Wang et al., 2022; Wang et al., 2020; Zhu et al., 2023; Ceciliani et al., 2020; Tang et al., 2024).

In addition to N-ε-methyl-L-lysine and ropinirole, several other metabolites identified in this study offer insights into the metabolic alterations underlying subclinical mastitis (SCM). Digalacturonic acid, elevated in both fecal and milk samples, likely reflects increased microbial pectin degradation and may indicate gut barrier disruption or microbial dysbiosis associated with systemic inflammation (Beukema et al., 2020). Acetylcarnitine, upregulated in serum and milk, is involved in fatty acid transport and energy metabolism. Its elevation suggests a metabolic shift toward lipid mobilization under inflammatory stress, consistent with patterns seen in preclinical mastitis states (Zandkarimi et al., 2018). Arachidonic acid, a precursor of pro-inflammatory eicosanoids, was positively associated with Succinivibrionaceae UCG-001, supporting its role as a mediator of host–microbe inflammatory interactions (Rinaldi et al., 2021).

These findings underscore the value of metabolite profiling in revealing host–microbe interactions and suggest that digalacturonic acid, acetylcarnitine, and arachidonic acid may serve as candidate biomarkers for SCM-related metabolic and immune disturbances. Metabolic pathway analysis revealed dysregulation in essential biosynthetic and degradation pathways, including amino acid and bile acid metabolism, galactose metabolism, and microbial metabolic networks. These systemic alterations reflect both host and microbial contributions to SCM pathology, further emphasizing the importance of gut and rumen microbial homeostasis in disease progression (Wang et al., 2021a; Saleh et al., 2022; Alessandri et al., 2023b).

16S rRNA-based microbiota analysis demonstrated distinct compositional shifts, with enrichment or depletion of specific genera linked to inflammation and metabolic dysregulation. Notably, alterations in *Succinivibrio*, *Succinivibrionaceae UCG-001*, *Alistipes*, and *Methanobrevibacter* were consistently associated with changes in metabolite levels. These functional predictions, based on 16S rRNA profiles, suggest a potential association between microbial changes and immune-related signaling. However, as these are computational inferences, they should not be interpreted as direct evidence of functional activity.

The observed correlations between metabolites and microbiota, particularly involving *Alistipes* and *Succinivibrionaceae UCG-001*, highlight potential axes of host–microbe metabolic crosstalk. Alistipes, known for its role in short-chain fatty acid production and inflammatory diseases, was inversely associated with key inflammatory metabolites (Wang et al., 2021c; Wang et al., 2022; Haxhiaj et al., 2022). The neuromodulatory compound ropinirole showed multiple associations across microbial genera, indicating possible microbiota-mediated neurometabolic effects during SCM (Couvillion et al., 2023; Hu et al., 2021). The inverse correlations between alpha diversity and inflammation-related metabolites support the hypothesis that microbial richness loss contributes to metabolic imbalance in SCM.

Our findings underscore the importance of sample-specific biomarker discovery. For instance, fecal bile acid metabolites demonstrated strong diagnostic potential, while serum and milk biomarkers may better reflect systemic inflammation. The distinct distribution patterns of ropinirole and N-ε-methyl-L-lysine across matrices highlight the value of multi-site sampling to enhance diagnostic precision.

The divergence of microbiota profiles between rumen fluid and feces further reinforces the complexity of SCM’s microbial ecology. These differences may stem from the distinct functional roles and microenvironments of these compartments. Elevated *Euryarchaeota* levels, linked to methane production and vitamin biosynthesis, suggest metabolic consequences that extend beyond the mammary gland and could impact feed efficiency and host metabolism (Mudaliar et al., 2017; Wang et al., 2021a). Although milk microbial communities are directly relevant to mastitis, we focused on fecal and rumen microbiota to capture gut-derived systemic influences and host–microbe metabolic interactions. These matrices provide more stable microbial signatures and lower susceptibility to environmental contamination. Practical constraints, including sequencing resources and sample throughput, also influenced this decision. Future studies integrating milk microbiome data will be essential to more comprehensively understand both local and systemic microbiota contributions to subclinical mastitis.

In practical terms, our results advocate for the incorporation of integrative metabolomic-microbiome monitoring in SCM surveillance programs. Such approaches could facilitate earlier detection, guide probiotic or dietary interventions, and inform precision health strategies to mitigate economic losses and improve herd productivity. Future studies should explore causality and validate these findings in larger cohorts to support clinical translation.

## 5 Conclusion

This study provides a comprehensive systems level characterization of subclinical mastitis (SCM) in dairy cows through the integration of metabolomic and microbiome data across multiple biological matrices. Our findings reveal that SCM is marked by subtle but pervasive immune activation, metabolic reprogramming, and microbial dysbiosis. Key biomarkers such as N-ε-methyl-L-lysine, digalacturonic acid, and ropinirole were identified consistently across matrices, offering promising candidates for non-invasive and multi-source detection of SCM. Furthermore, while ropinirole was validated through targeted analysis, its synthetic origin and uncertain source highlight the need for caution. Its inclusion as a biomarker is premature without further investigation into potential exogenous contamination.

The altered microbial signatures, particularly involving *Succinivibrionaceae UCG-001* and *Alistipes*, and their correlations with inflammatory and neuromodulatory metabolites, highlight the intricate host–microbiome interactions underlying disease progression. Functional pathway analysis further supports the involvement of immune signaling, energy metabolism, and microbial biosynthetic activity in SCM pathophysiology.

Collectively, this multi-omics approach advances our mechanistic understanding of SCM and lays the groundwork for early diagnostic biomarkers and precision-targeted interventions. Incorporating these findings into herd health management strategies could significantly enhance disease monitoring, reduce productivity losses, and improve animal welfare.

Future research should focus on longitudinal validation of identified biomarkers and exploration of causative links to further support clinical translation and intervention development.

## Data Availability

The original contributions presented in the study are publicly available. This data can be found in at: https://www.ncbi.nlm.nih.gov/sra/PRJNA1190230.

## Glossary

ANOVA: Analysis of variance
CBC: Complete blood count
DHI: Dairy herd improvement
FDR: False discovery rate
FTIR: Fourier transform infrared
Hb: Hemoglobin
HCT: Hematocrit
KEGG: Kyoto Encyclopedia of Genes and Genomes
LC-MS: Liquid chromatography-mass spectrometry
MS: Mass spectrometry
NMDS: Non-Metric Multidimensional Scaling
OPLS-DA: Orthogonal partial least squares discriminant analysis
PCA: Principal component analysis
PLT: Platelet count
PLS-DA: Partial least squares discriminant analysis
RBC: Red blood cell
SCM: Subclinical mastitis
SCC: Somatic cell count
SEM: Standard error of the mean
UPLC: Ultra-performance liquid chromatography
VIP: Variable importance in projection
WBC: White blood cell

## References

[ref1] Abed A. Menshawy A. Zeinhom M. Hossain D. Khalifa E. Wareth G.. (2021). Subclinical mastitis in selected bovine dairy herds in North Upper Egypt: assessment of prevalence, causative bacterial pathogens, antimicrobial resistance and virulence-associated genes. Microorganisms 9:1175. doi: 10.3390/microorganisms9061175, PMID: 34072543 PMC8229104

[ref2] Alessandri G. Sangalli E. Facchi M. Fontana F. Mancabelli L. Donofrio G.. (2023a). Metataxonomic analysis of milk microbiota in the bovine subclinical mastitis. FEMS Microbiol. Ecol. 99:fiad136. doi: 10.1093/femsec/fiad136, PMID: 37880979

[ref3] Alessandri G. Sangalli E. Facchi M. Fontana F. Mancabelli L. Donofrio G.. (2023b). Metagenomic analysis of milk microbiota in the bovine subclinical mastitis. *bioRxiv*. Available online at: 10.1101/2023.05.09.539964. [Epub ahead of preprint] 37880979

[ref4] Alvanou M. V. Loukovitis D. Melfou K. Giantsis I. A. (2024). Utility of dairy microbiome as a tool for authentication and traceability. Open Life Sci. 19:20220983. doi: 10.1515/biol-2022-0983, PMID: 39479351 PMC11524395

[ref6] Argaw A. (2016). Review on epidemiology of clinical and subclinical mastitis on dairy cows. Food Sci. Qual. Manag. 52, 56–65.

[ref16] Beukema M. Faas M. M. de Vos P. (2020). The effects of different dietary fiber pectin structures on the gastrointestinal immune barrier: impact via gut microbiota and direct effects on immune cells. Exp. Mol. Med. 52, 1364–1376. doi: 10.1038/s12276-020-0449-2 32908213 PMC8080816

[ref8] Ceciliani F. Audano M. Addis F. Mitro N. Caruso D. Lecchi C.. (2020). The subclinical non-aureus staphylococcal mastitis in dairy cows: a lipidomics approach. J. Anim. Sci. 98, 82–83. doi: 10.1093/jas/skaa278.151

[ref9] Couvillion S. P. Mostoller K. E. Williams J. E. Pace R. M. Stohel I. L. Peterson H. K.. (2023). Interrogating the role of the milk microbiome in mastitis in the multi-omics era. Front. Microbiol. 14:1105675. doi: 10.3389/fmicb.2023.1105675, PMID: 36819069 PMC9932517

[ref10] Demil E. Teshome L. Kerie Y. Habtamu A. Kumilachew W. Andualem T.. (2022). Prevalence of subclinical mastitis, associated risk factors and antimicrobial susceptibility of the pathogens isolated from milk samples of dairy cows in Northwest Ethiopia. Prev. Vet. Med. 205:105680. doi: 10.1016/j.prevetmed.2022.105680, PMID: 35691136

[ref11] Eleodoro J. I. Headley S. A. Zanoni A. P. K. Giordano L. G. P. Fagnani R. (2022). Most common pathogens from cows with subclinical mastitis in northwestern Paraná, Southern Brazil and their antimicrobial susceptibility. Semin. Ciênc. Agrár. 43, 2385–2394. doi: 10.5433/1679-0359.2022v43n5p2385

[ref12] El-Tawab A. A. Hofy F. Ammar A. Sleim M. Salem H. (2016). Prevalence and antimicrobial susceptibility pattern of *Staphylococcus aureus* isolated from dairy cattle’s subclinical mastitis in EL-Sharkia Governorate. Benha Vet. Med. J. 30, 11–19. doi: 10.21608/BVMJ.2016.31333

[ref13] Farabi A. A. Hossain H. Brishty K. Rahman M. Rahman M. Siddiqui M. S. I.. (2024). Prevalence, risk factors, and antimicrobial resistance of *Staphylococcus* and *Streptococcus* species isolated from subclinical bovine mastitis. Foodborne Pathog. Dis. 22, 467–476. doi: 10.1089/fpd.2024.0097 39479784

[ref14] Haxhiaj K. Wishart D. Ametaj B. (2022). Mastitis: what it is, current diagnostics, and the potential of metabolomics to identify new predictive biomarkers. Dairy 3, 626–651. doi: 10.3390/dairy3040050

[ref15] Hu H. Fang Z. Mu T. Wang Z. Ma Y. Ma Y. (2021). Application of metabolomics in diagnosis of cow mastitis: a review. Front. Vet. Sci. 8:747519. doi: 10.3389/fvets.2021.747519, PMID: 34692813 PMC8531087

[ref17] Khasapane N. Koos M. Nkhebenyane S. Khumalo Z. Ramatla T. A. Thekisoe O. (2024). Detection of *Staphylococcus* isolates and their antimicrobial resistance profiles and virulence genes from subclinical mastitis cattle milk using MALDI-TOF MS, PCR and sequencing in Free State Province, South Africa. Animals 14:154. doi: 10.3390/ani14010154, PMID: 38200885 PMC10778211

[ref7] Kumari T. Bhakat C. Choudhary R. K. (2018). A review on sub clinical mastitis in dairy cattle. Int. J. Pure App. Biosci. 6, 1291–1299. doi: 10.18782/2320-7051.6173

[ref18] Michira L. Kagira J. Maina N. Waititu K. Kiboi D. Ongera E.. (2023). Prevalence of subclinical mastitis, associated risk factors and antimicrobial susceptibility pattern of bacteria isolated from milk of dairy cattle in Kajiado Central sub-county, Kenya. Vet. Med. Sci. 9, 2885–2892. doi: 10.1002/vms3.1291, PMID: 37792167 PMC10650227

[ref19] Mudaliar M. Thomas F. Eckersall P. D. (2017). “Omic approaches to a better understanding of mastitis in dairy cows” in Omics applications in animal science (Cham: Springer), 139–183.

[ref20] Qolbaini E. N. Khoeri M. M. Salsabila K. Paramaiswari W. Tafroji W. Artika I.. (2021). Identification and antimicrobial susceptibility of methicillin-resistant *Staphylococcus aureus*-associated subclinical mastitis isolated from dairy cows in Bogor, Indonesia. Vet. World 14, 1180–1184. doi: 10.14202/vetworld.2021.1180-1184, PMID: 34220119 PMC8243663

[ref21] Rinaldi M. Li R. W. Capuco A. V. (2021). Inflammation and arachidonic acid signaling in dairy cow mastitis. Animals 11:2956. doi: 10.3390/ani11102956 34679977 PMC8532900

[ref22] Ruegg P. L. (2012). Mastitis in dairy cows. Vet. Clin. North Am. Food Anim. Pract. 28, xi–xii. doi: 10.1016/j.cvfa.2012.04.003, PMID: 22664214

[ref23] Saleh N. Allam T. Omran A. Mohamed A. (2022). A review of the subclinical mastitis in cattle with special reference to the new approaches of its diagnosis and control. J. Curr. Vet. Res. 4, 17–26. doi: 10.21608/jcvr.2022.240863

[ref24] Tang R. Yang W. Song J. Xiang K. Li S. Zhao C.. (2024). The rumen microbiota contributed to the development of mastitis induced by subclinical ketosis. Microb. Pathog. 187:106509. doi: 10.1016/j.micpath.2023.106509 38185451

[ref25] Tassew A. (2017). Isolation, identification and antimicrobial resistance profile of *Staphylococcus aureus* and occurrence of methicillin resistant *S. aureus* isolated from mastitic lactating cows in and around Assosa town, Benishangul Gumuz Region, Ethiopia. Adv. Life Sci. Technol. 60, 23–32. doi: 10.28933/ijar-2017-09-2302

[ref26] Tassew A. Negash M. Demeke A. Feleke A. Tesfaye B. Sisay T. (2016). Isolation, identification and drug resistance patterns of methicillin resistant *Staphylococcus aureus* from mastitic cows milk from selected dairy farms in and around Kombolcha, Ethiopia. J. Vet. Med. Anim. Health 8, 1–10. doi: 10.5897/JVMAH2015.0422

[ref27] Thomas F. Mudaliar M. Tassi R. Thomas F. C. McNeilly T. N. Burchmore R.. (2016). Mastitomics, the integrated omics of bovine milk in an experimental model of *Streptococcus uberis* mastitis: 3. Untargeted metabolomics. Mol. BioSyst. 12, 2762–2769. doi: 10.1039/c6mb00289g, PMID: 27412568

[ref28] Tommasoni C. Fiore E. Lisuzzo A. Gianesella M. (2023). Mastitis in dairy cattle: on-farm diagnostics and future perspectives. Animals 13:2538. doi: 10.3390/ani13152538, PMID: 37570346 PMC10417731

[ref29] Tong J. Zhang H. Zhang Y. H. Xiong B. Jiang L. (2019). Microbiome and metabolome analyses of milk from dairy cows with subclinical *Streptococcus agalactiae* mastitis—potential biomarkers. Front. Microbiol. 10:2547. doi: 10.3389/fmicb.2019.02547, PMID: 31781063 PMC6851174

[ref30] Wang L. Haq S. U. Shoaib M. He J. Guo W. Wei X.. (2024). Subclinical mastitis in small-holder dairy herds of Gansu Province, Northwest China: prevalence, bacterial pathogens, antimicrobial susceptibility, and risk factor analysis. Microorganisms 12:2643. doi: 10.3390/microorganisms12122643, PMID: 39770845 PMC11727839

[ref31] Wang Y. Nan X. Zhao Y. Jiang L. Wang M. Wang H.. (2021a). Rumen microbiome structure and metabolites activity in dairy cows with clinical and subclinical mastitis. J. Anim. Sci. Biotechnol. 12:36. doi: 10.1186/s40104-020-00543-1, PMID: 33557959 PMC7869221

[ref32] Wang Y. Nan X. Zhao Y. Jiang L. Wang H. Zhang F.. (2022). Changes in the profile of fecal microbiota and metabolites as well as serum metabolites and proteome after dietary inulin supplementation in dairy cows with subclinical mastitis. Front. Microbiol. 13:809139. doi: 10.3389/fmicb.2022.809139, PMID: 35479637 PMC9037088

[ref33] Wang Y. Nan X. Zhao Y. Jiang L. Wang H. Zhang F.. (2021b). Dietary supplementation of inulin ameliorates subclinical mastitis via regulation of rumen microbial community and metabolites in dairy cows. Microbiol. Spectr. 9:e00105-21. doi: 10.1128/Spectrum.00105-21, PMID: 34494854 PMC8557905

[ref34] Wang Y. Nan X. Zhao Y. Jiang L. Wang H. Zhang F.. (2021c). Consumption of supplementary inulin modulates milk microbiota and metabolites in dairy cows with subclinical mastitis. Appl. Environ. Microbiol. 88:e0205921. doi: 10.1128/aem.02059-21 34936838 PMC8942464

[ref35] Wang Y. Nan X. Zhao Y. Wang H. Wang M. Jiang L.. (2020). Coupling 16s rDNA and untargeted mass spectrometry for milk microbial composition and metabolites from dairy cows with clinical and subclinical mastitis. J. Agric. Food Chem. 68, 8737–8746. doi: 10.1021/acs.jafc.0c03738 32633125

[ref36] Xi X. Kwok L. Y. Wang Y. Ma C. Mi Z. Zhang H. (2017). Ultra-performance liquid chromatography-quadrupole-time of flight mass spectrometry MSE-based untargeted milk metabolomics in dairy cows with subclinical or clinical mastitis. J. Dairy Sci. 100, 4884–4896. doi: 10.3168/jds.2016-11939, PMID: 28342601

[ref37] Zandkarimi F. Vanegas J. Fern X. Z. Maier C. S. Bobe G. (2018). Metabotypes with elevated protein and lipid catabolism and inflammation precede clinical mastitis in prepartal transition dairy cows. J. Dairy Sci. 101, 5531–5548. doi: 10.3168/jds.2017-13977, PMID: 29573799 PMC6431296

[ref38] Zhu C. Zhang Q. Zhao X. Yang Z. Yang F. Yang Y.. (2023). Metabolomic analysis of multiple biological specimens (feces, serum, and urine) by 1H-NMR spectroscopy from dairy cows with clinical mastitis. Animals 13:741. doi: 10.3390/ani13040741, PMID: 36830529 PMC9952568

[ref39] Zhu C. Zhao Y. Yang F. Zhang Q. Zhao X. Yang Z.. (2024). Microbiome and metabolome analyses of milk and feces from dairy cows with healthy, subclinical, and clinical mastitis. Front. Microbiol. 15:1374911. doi: 10.3389/fmicb.2024.1374911, PMID: 38912351 PMC11191547

