# Supplementary material for: Cross-matrix multi-omics profiling identifies host–microbe interactions and diagnostic signatures in bovine subclinical mastitis
Source: Front Microbiol. 2025 Aug 5;16:1613949. doi: 10.3389/fmicb.2025.1613949 (PMC12369410; doi:10.3389/fmicb.2025.1613949)
Supplement: Supplementary file 1 [file Data_Sheet_1.docx]

**Materials and Methods**

**Metabolite Extraction and LC-MS/MS Analysis**

**Sample Collection and Preparation**

Milk, fecal, rumen fluid, and plasma samples were processed using a standardized protocol to ensure reliable and reproducible metabolite profiling. Milk samples were centrifuged at 4 °C to remove the cream layer, followed by protein precipitation. Fecal samples were homogenized, and rumen fluid samples were filtered to eliminate particulates prior to extraction. Plasma samples were thawed on ice and directly subjected to extraction.

**Protein Precipitation and Metabolite Extraction**

A 100 μL aliquot of each sample was mixed with 400 μL of ice-cold methanol/acetonitrile (1:1, v/v) to precipitate proteins and extract metabolites. Samples were vortexed for 30 s, incubated on ice for 10 min, and centrifuged at 14,000 × g for 15 min at 4 °C. Supernatants were transferred to clean tubes and evaporated to dryness using a vacuum centrifugal concentrator. The dried extracts were reconstituted in 100 μL of acetonitrile/water (1:1, v/v) and filtered through 0.22 μm PTFE filters before LC-MS analysis.

**Quality Control (QC) Strategy**

A pooled QC sample was prepared by mixing 10 μL from each individual extract. QC samples were injected at regular intervals (every five samples) throughout the run to monitor analytical stability, including retention time consistency and signal drift.

**UHPLC-QTOF-MS Conditions**

**Chromatographic Separation**

Analyses were performed using an Agilent 1290 Infinity UHPLC system coupled with a TripleTOF 6600 mass spectrometer (AB Sciex). Separation was achieved on a Waters ACQUITY UPLC BEH Amide column (2.1 × 100 mm, 1.7 μm) under hydrophilic interaction liquid chromatography (HILIC) conditions.

Mobile Phase A: 25 mM ammonium acetate and 25 mM ammonium hydroxide in H₂O Mobile Phase B: Acetonitrile

Gradient Elution Program: 0.0–1.0 min: 85%; 1.0–12.0 min: linear decrease to 65%; 12.0–12.1 min: step decrease to 40%; 12.1–16.1 min: hold at 40%; 16.1–16.2 min: increase to 85%; 16.2–21.2 min: re-equilibration at 85%

**Mass Spectrometry Parameters**

Data were acquired in both positive and negative ion modes.Ion Source Parameters: Curtain gas: 30 psi; Ion source gas 1 and 2: 60 psi; Source temperature: 600 °C; IonSpray voltage floating (ISVF): ±5500 V.Acquisition Settings: MS1 range: m/z 60–1000; scan time: 0.20 s; MS/MS (IDA): m/z 25–1000; accumulation time: 0.05 s; Collision energy: 35 ± 15 eV; Declustering potential: ±60 V; Max candidate ions: 10; Isotope exclusion: ±4 Da.

**Data Processing and Statistical Analysis**

**Data Conversion and Preprocessing**

Raw spectral data were converted to mzXML format using MSConvert (ProteoWizard). Peak picking, alignment, and grouping were conducted using the XCMS package in R. Peak Detection Method: centWave m/z tolerance = 10 ppm; Peak width = c (10, 60); Prefilter = c (10, 100); Grouping: bw = 5, mzwid = 0.025, minfrac = 0.5.

**Feature Annotation and Normalization**

Feature annotation for isotopes and adducts was performed using CAMERA. Features with >50% non-zero values in at least one group were retained. Missing values were imputed using the K-nearest neighbor (KNN) algorithm. Total ion current (TIC) normalization was applied, and outliers were removed.

**Metabolite Identification**

Putative metabolite identities were assigned by matching accurate m/z values (mass error <10 ppm) and MS/MS spectra against an in-house spectral library of authenticated standards.

**Statistical Analysis**

All analyses were conducted in R (v4.0.3). Data were log10-transformed, mean-centered, and scaled to unit variance prior to multivariate analysis. Unsupervised: PCA; Supervised: OPLS-DA with 200-permutation validation; Feature selection: VIP >1.0, t-test p < 0.05; Interpretation: log2 fold change, KEGG pathway enrichment, FDR correction.

**16S rRNA Gene Sequencing and Microbiome Analysis**

**Sample Collection and DNA Extraction**

Fecal samples were collected aseptically from the rectum, and rumen fluid was obtained via rumenocentesis or cannula. Samples were immediately placed on ice and stored at –80 °C. Genomic DNA was extracted using mechanical bead-beating combined with chemical lysis. Purity was assessed using NanoDrop (A260/A280 and A260/A230), and concentrations were quantified using Qubit.

**PCR Amplification and Library Preparation**

The V4 region of the 16S rRNA gene was amplified using primers 515F/806R. Negative controls were included. Amplicons were purified, dual-index barcoded, and pooled equimolarly.

**Sequencing and Bioinformatics Analysis**

Sequencing was performed on an Illumina platform (2 × 250 bp). Quality filtering, chimera removal, and sequence denoising were performed using the DADA2 pipeline. Taxonomic assignment was based on curated reference databases. Alpha-diversity: Shannon, Chao1; Beta-diversity: Weighted UniFrac, NMDS, PCoA; Group differences: PERMANOVA; Functional prediction: PICRUSt or Tax4Fun
