# Supplementary figures and images for: Cross-matrix multi-omics profiling identifies host–microbe interactions and diagnostic signatures in bovine subclinical mastitis
Source: Front Microbiol. 2025 Aug 5;16:1613949. doi: 10.3389/fmicb.2025.1613949 (PMC12369410; doi:10.3389/fmicb.2025.1613949)

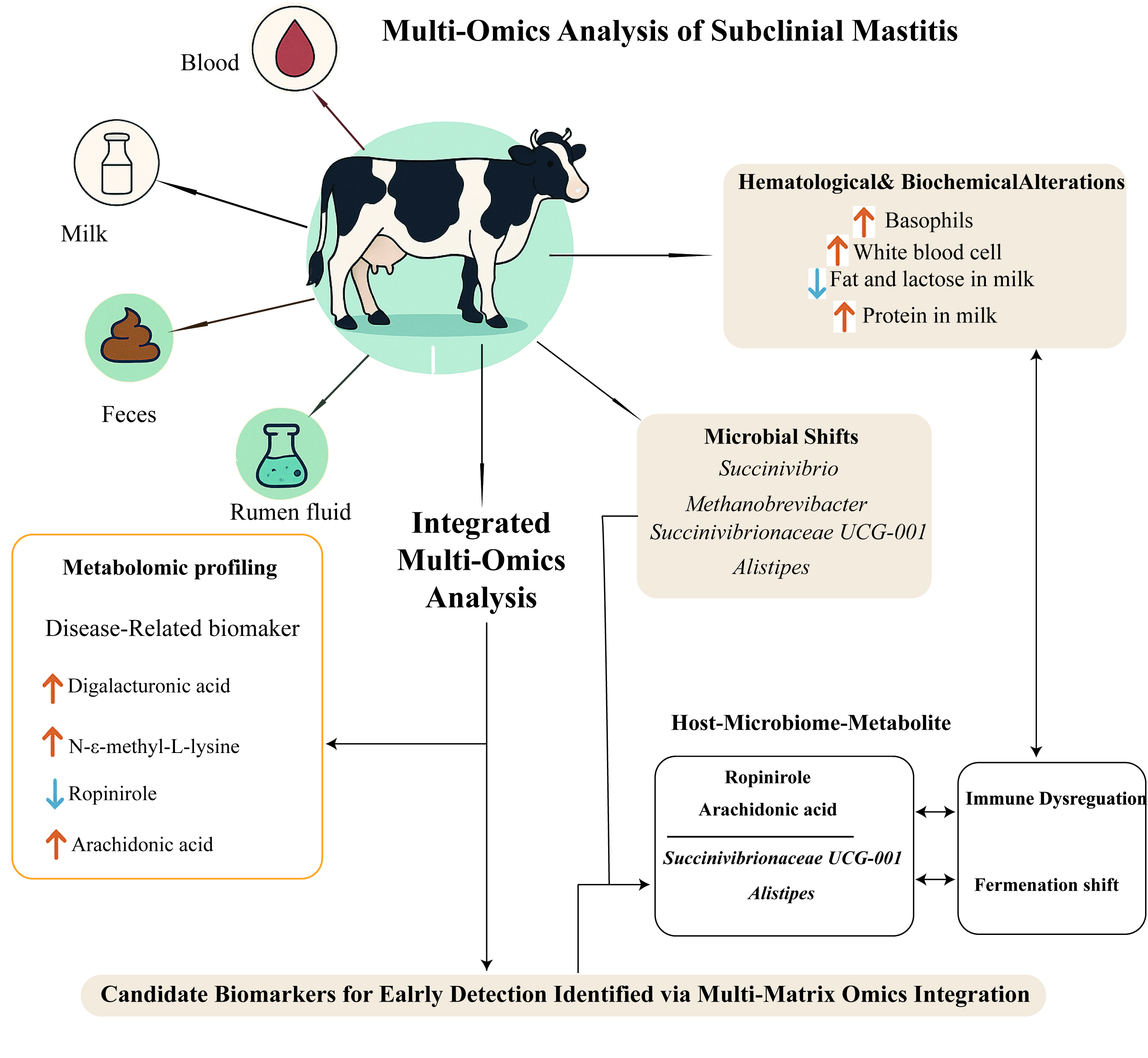

Supplement: Supplementary file 2 [file Image_1.tif]

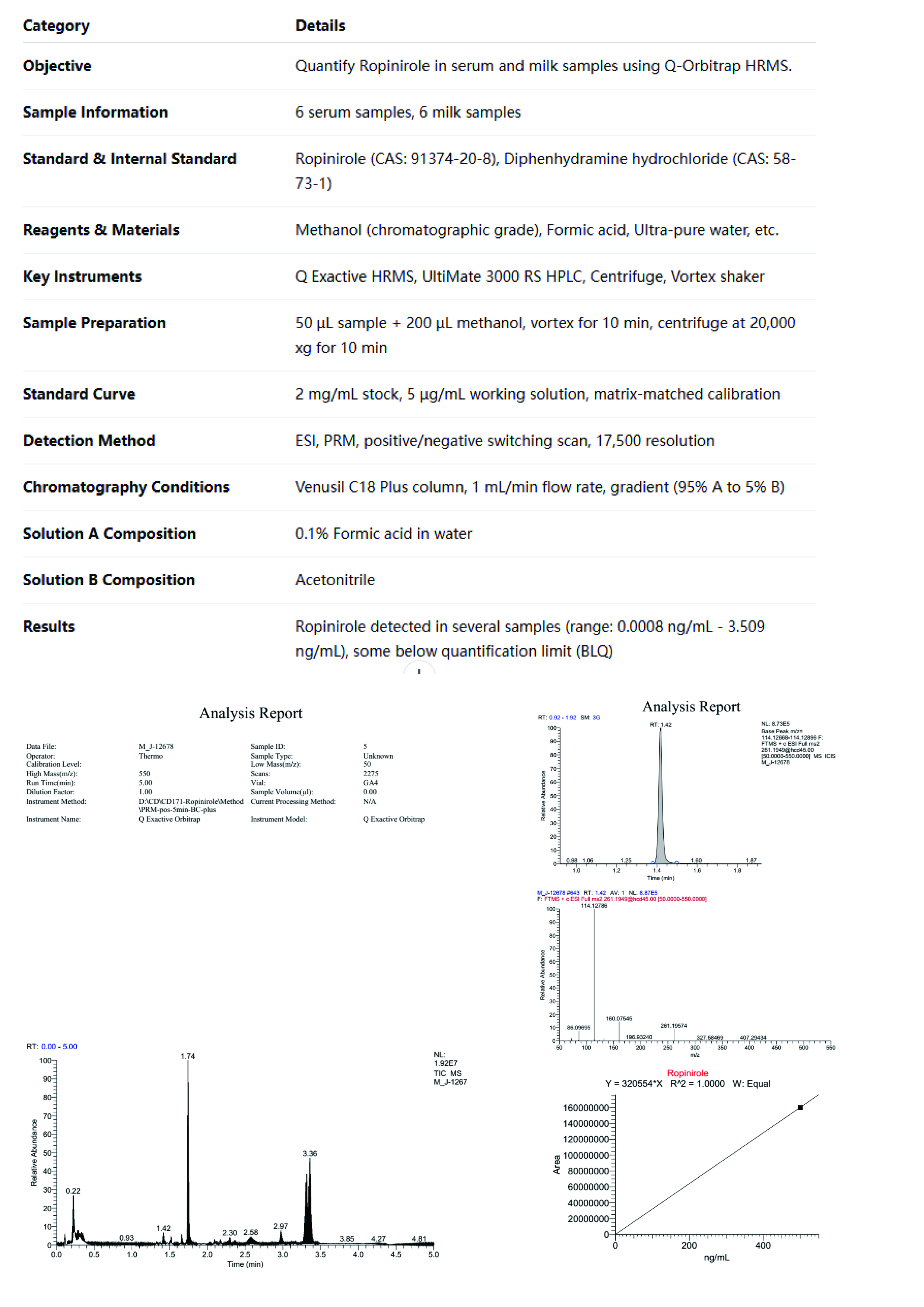

Supplement: Supplementary file 3 [file Image_2.tif]
