# Supplementary material for: Cross-matrix multi-omics profiling identifies host–microbe interactions and diagnostic signatures in bovine subclinical mastitis
Source: Front Microbiol. 2025 Aug 5;16:1613949. doi: 10.3389/fmicb.2025.1613949 (PMC12369410; doi:10.3389/fmicb.2025.1613949)
Supplement: Supplementary file 4 [file Table_1.docx]

**Supplementary Table S1. Results of PERMANOVA for metabolome**

| Sample Type | Groups Compared | Distance Metric | Permutations | F-value | R² | p-value |
| --- | --- | --- | --- | --- | --- | --- |
| Serum | SCM vs. Healthy | Bray–Curtis | 999 | 2.432 | 0.198 | 0.019 |
| Milk | SCM vs. Healthy | Bray–Curtis | 999 | 2.851 | 0.223 | 0.007 |
| Feces | SCM vs. Healthy | Bray–Curtis | 999 | 2.124 | 0.175 | 0.022 |
| Rumen | SCM vs. Healthy | Bray–Curtis | 999 | 2.173 | 0.184 | 0.015 |
